# Hyperpolarization-Activated Current, *I*_*f*_, in Mathematical Models of Rabbit Sinoatrial Node Pacemaker Cells

**DOI:** 10.1155/2013/872454

**Published:** 2013-07-08

**Authors:** Arie O. Verkerk, Ronald Wilders

**Affiliations:** Department of Anatomy, Embryology and Physiology, Academic Medical Center, University of Amsterdam, P.O. Box 22700, 1100 DE Amsterdam, The Netherlands

## Abstract

A typical feature of sinoatrial (SA) node pacemaker cells is the presence of an ionic current that activates upon hyperpolarization. The role of this hyperpolarization-activated current, *I*_*f*_, which is also known as the “funny current” or “pacemaker current,” in the spontaneous pacemaker activity of SA nodal cells remains a matter of intense debate. Whereas some conclude that *I*_*f*_ plays a fundamental role in the generation of pacemaker activity and its rate control, others conclude that the role of *I*_*f*_ is limited to a modest contribution to rate control. The ongoing debate is often accompanied with arguments from computer simulations, either to support one's personal view or to invalidate that of the antagonist. In the present paper, we review the various mathematical descriptions of *I*_*f*_ that have been used in computer simulations and compare their strikingly different characteristics with our experimental data. We identify caveats and propose a novel model for *I*_*f*_ based on our experimental data.

## 1. A Funny Current

The sinoatrial node (SA node) is the normal pacemaker of the mammalian heart and generates the electrical impulse for the regular, rhythmic contraction of the heart. The intrinsic pacemaker activity, or spontaneous electrical activity, of an SA nodal pacemaker cell is based on the spontaneous diastolic depolarization that depolarizes the cell towards the action potential threshold. During this diastolic depolarization phase, there is a tiny net inward current across the cell membrane of no more than a few picoamps in amplitude. Animal studies, almost exclusively carried out on cells isolated from rabbit heart, have learned that this net inward current is the result of a complex interaction of multiple inward and outward ion currents, including a hyperpolarization-activated current of mixed ionic nature, known as “funny current,” *I*_*f*_, as reviewed elsewhere [1–9]. In line with its activation at hyperpolarized membrane potentials [10], thus generating an inward current during diastole, its enhancement by direct binding of cyclic AMP (cAMP) [11], and its principal presence in primary [12] and secondary pacemakers [13, 14], *I*_*f*_ is traditionally also named “pacemaker current.” Of note, the pore-forming subunits of the *I*_*f*_ channel are encoded by members of the *HCN* gene family, with members *HCN1-4* (see [8] and primary references cited therein). HCN channels are not only expressed in the heart but also in the brain. In neuroscience, the HCN current is usually designated *I*_*h*_.

In recent years, *I*_*f*_ has regained interest in several fields. First, *I*_*f*_ has become a target for pharmacological reduction of heart rate, which may be beneficial for heart failure patients. This reduction is achieved through the specific *I*_*f*_ blocker ivabradine, which has become available for clinical use, and represents a new approach in selective heart rate reduction [15]. Second, mutations in the *HCN4* gene, encoding the major HCN isoform of the human SA node [16], have been associated with hereditary SA nodal dysfunction in several families [17–23]. Third, HCN channels are used in engineering a biological pacemaker, as summarized in numerous review papers, for example, [24–29]. In all of these fields, an appropriate quantitative model of the electrical activity of *I*_*f*_ is a desirable tool.

It should be noted that *I*_*f*_ depends on intracellular calcium levels, and conversely, through the mutual interactions between *I*_*f*_ amplitude, spontaneous firing frequency and intracellular Ca^2+^ cycling [30, 31]. Thus, a blockade of *I*_*f*_ by Cs^+^ [30] or ivabradine [31] does not only affect firing frequency, but also intracellular Ca^2+^ cycling. In the interactions between the “membrane clock” (composed of voltage-dependent sarcolemmal currents and also designated “M clock,” “voltage clock,” or “ion channel clock”) and the “calcium clock” or “Ca^2+^ clock” (composed of tightly coupled sarcoplasmic reticulum Ca^2+^ cycling molecules together with the electrogenic sodium-calcium exchanger), cAMP plays an important role [9]. It is, therefore, important to make measurements on  *I*_*f*_  with the amphotericin-perforated patch configuration of the patch-clamp technique to avoid the dialyzing effects of the common whole-cell configuration. Instead of rupturing the membrane as in the whole-cell patch clamp configuration, amphotericin B is used to gain electrical access to the cell [32]. This substance makes minute holes in the membrane that allow the passage of small monovalent ions, thus leaving the cytosolic composition intact. In particular, the intracellular Ca^2+^ and cAMP levels are preserved.

Differences in recording conditions like the aforementioned patch clamp configurations may readily explain part of the variability in experimental data on *I*_*f*_ between laboratories. A further source of variability is observed in measurements on HCN channels in heterologous expression systems like HEK-293 cells [8]. Here, part of the variability can be attributed to differences in the expression level of members of the MinK family of single transmembrane spanning proteins, which are encoded by the *KCNE* gene family and can act as *β*-subunits for the HCN family of pore-forming *α*-subunits [33–35]. In particular, the MinK-related peptide 1 (MiRP1, encoded by *KCNE2*), with high mRNA levels in the rabbit SA node [33], may associate with HCN1, HCN2, and HCN4 and modulate the HCN channel expression and kinetics.

Originally, *I*_*f*_ was termed “funny current” because of its atypical characteristics, including its slow activation upon hyperpolarization rather than depolarization [10], its direct activation by cAMP [11], and its highly selective permeability to both Na^+^ and K^+^ ions [36] with a *P*_Na_ : *P*_K_ permeability ratio of approximately 1 : 4 under physiological conditions [37]. As a result of its mixed ionic nature, *I*_*f*_ exhibits a reversal potential of *≈*−30 mV if corrected for the liquid junction potential [38–40]. Thus, *I*_*f*_ is an inward current carried by sodium ions at diastolic membrane potentials. However, this Na^+^ current is critically dependent on the presence of extracellular K^+^ ions. It increases with increasing extracellular K^+^ concentration, as does the *P*_Na_ : *P*_K_ ratio, which saturates near the physiological extracellular K^+^ concentration of *≈*5 mM [41]. More recently, it was shown that *I*_*f*_ channels are also permeable, albeit to it small extent, to Ca^2+^ ions [42, 43], with the Ca^2+^ flux contributing to *≈*0.5% of the current produced by *I*_*f*_ [42]. However, the functional relevance of this permeability to Ca^2+^ remains unclear [44].

Another characteristic feature of HCN channels, and thus possibly of *I*_*f*_, is their ability to undergo a “mode shift” in their voltage gating. This mode shift or “voltage hysteresis” has been studied for HCN1, HCN2, and HCN4 channels that were heterologously expressed in *Xenopus *oocytes or mammalian COS-7 or HEK-293 cells [45–49]. The voltage hysteresis is clearly present in HCN1 channels [45, 46, 48] but less pronounced in HNC2 channels [46, 47]. Whereas both Azene et al. [46] and Elinder et al. [47] concluded that voltage hysteresis is absent or almost absent in HNC4 channels, Xiao et al. [49] reported a clear hysteresis. Given that in most species, including rabbit, HCN4 is by far the most abundant HCN isoform in the SA node [8], it remains to be elucidated whether voltage hysteresis of *I*_*f*_ plays a functional role in cardiac pacemaker activity. However, voltage hysteresis may prove important in fine-tuning the firing frequency of gene- and cell-based biological pacemakers, in particular if these make use of HCN1 or HCN2 [46].

Despite the numerous experimental studies, the contribution of *I*_*f*_ to SA nodal pacemaker activity has been and still is a matter of, often intense, debate, particularly in relation to the calcium clock [50–64]. A complicating factor in this ongoing debate is created by the slow activation kinetics and negative activation profile of *I*_*f*_ relative to the time scale and voltage range of SA nodal diastolic depolarization, which makes only a small number of  *I*_*f*_  channels activated during diastolic depolarization. In a total of 12 mathematical models of the pacemaker activity of rabbit SA nodal cells published between 1982 and 2003 quantitatively widely different mechanisms underlie spontaneous diastolic depolarization, as demonstrated by the 0.9–30% increase in cycle length upon block of *I*_*f*_ [65]. It is, therefore, not surprising that computer simulations, albeit with “updated” models, have not only been used to support a limited role for *I*_*f*_ [66] but also to underscore that *I*_*f*_ is “the major inward diastolic ionic current” [67].

## 2. Experimental Data

**Figure 1:**
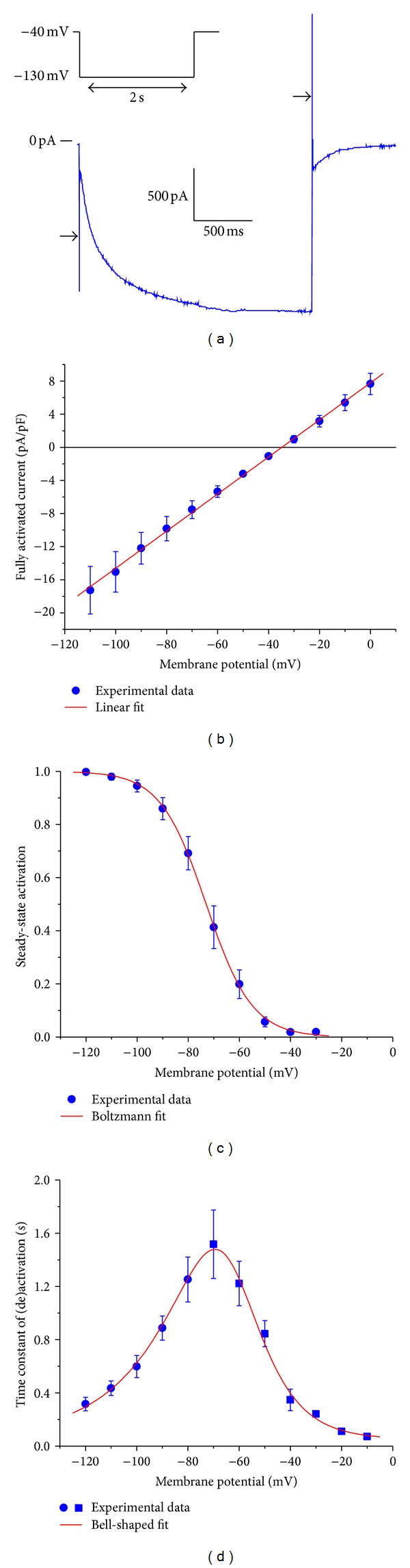
Characteristics of the hyperpolarization-activated current (*I*_*f*_) in single pacemaker cells isolated from the rabbit sinoatrial node. (a) Current trace (blue) in response to a voltage clamp step from a holding potential of −40 mV to a test potential of −130 mV (inset). *I*_*f*_ activates during the 2-s step (step current) and deactivates during the subsequent step to −40 mV (tail current). Horizontal arrows indicate the capacitive transient. (b) Fully activated current, normalized to membrane capacitance, as derived from the tail current amplitude at potentials ranging between −110 and 0 mV. The red line is the linear fit to the experimental data. (c) Normalized steady-state activation derived from the tail current amplitude observed upon test potentials between −120 and −30 mV. The red curve is the Boltzmann fit to the experimental data. (d) Time constant of *I*_*f*_ activation (filled circles) and deactivation (filled squares). The red curve is a bell-shaped fit to the experimental data (see text). All membrane potential values are corrected for the estimated liquid junction potential.

Figure 1 shows experimental data on  *I*_*f*_  in isolated rabbit SA nodal myocytes obtained at physiological temperature using the amphotericin-perforated patch-clamp technique under voltage clamp conditions. To minimize contamination by K^+^ and Ca^2+^ currents, data were recorded in the presence of 1 mM BaCl_2_, 5 *μ*M E4031, 5 *μ*M chromanol 293b, 0.5 mM 4-aminopyridine, 5 *μ*M nifedipine, and 40 *μ*M NiCl_2_ in the bath solution. The concentrations of K^+^ and Na^+^ in the bath solution were 5.4 and 140 mM, respectively, whereas those in the pipette solution were 145 and 10 mM, respectively. Of note, the data of Figure 1 are consistent with the data that we collected in previous studies under similar conditions [40, 68].

As an example of a voltage clamp trace, Figure 1(a) shows the membrane current in response to a voltage clamp step to −130 mV from a holding potential of −40 mV. In this example, the recorded current was not corrected for the capacitive transient that accompanies a voltage clamp step (Figure 1(a), arrows). At the end of the 2-s step to −130 mV, *I*_*f*_ is fully activated. From the “tail current” that is observed upon stepping back from −130 mV to potentials ranging between −110 and 0 mV (−40 mV in case of Figure 1(a)), one can derive the fully activated current-voltage relationship of Figure 1(b), in which the recorded current is normalized to membrane capacitance and thus expressed in pA/pF. From the linear fit to the data of Figure 1(b), assuming an ohmic current-voltage relationship, one obtains a fully activated *I*_*f*_ conductance of 0.224 nS/pF and an *I*_*f*_ reversal potential of −34.8 mV. Together with the sodium and potassium concentrations in the pipette and the bath, this reversal potential yields a *g*_*f*,Na_ : *g*_*f*,K_ ratio of 0.491 and a *P*_Na_ : *P*_K_ permeability ratio, according to the Goldman equation, of 1 : 4.3, in line with aforementioned permeability ratio of 1 : 4 under physiological conditions [37].

If the tail current associated with test potentials between −120 and −30 mV is normalized to the tail current associated with the step to −130 mV, at which *I*_*f*_ is fully activated, one determines the voltage dependence of activation, that is, the steady-state activation of *I*_*f*_ at a series of membrane potentials (Figure 1(c)). The red curve in Figure 1(c) results from the Boltzmann equation:

(1) $$\begin{array}{c} y_{\infty }=\frac{1}{\left\{1+\exp\left[\left(V_{m}-V_{0.5}\right)/k\right]\right\}}, \end{array}$$

in which *y*_*∞*_ is the degree of steady-state activation, *V*_*m*_ is the membrane potential, *V*_0.5_ is the half-activation voltage, and *k* is the slope factor. The fitting procedure yielded *V*_0.5_ and *k* values of −73.0 and 9.0 mV, respectively.

Ignoring the variable initial delay in *I*_*f*_ (de)activation, the *I*_*f*_ current traces closely resemble a monoexponential process, both during activation and deactivation (cf. Figure 1(a)), which at a given membrane potential can be well fitted using a single time constant *τ*. The resulting time constant data are shown in Figure 1(d). The red bell-shaped curve in Figure 1(d) represents

(2) τ=0.05+[75.8×exp⁡(0.083×Vm)     +0.0233×exp⁡(−0.043×Vm)]−1,

in which *τ* is the time constant of (de)activation in *s* and *V*_*m*_ is the membrane potential in mV. It is important to note that the fitted curve levels off at 50 ms for membrane potentials >−10 mV, in accordance with the experimental observation that *I*_*f*_ deactivation is fast but certainly not instantaneous at depolarized potentials [39, 69–71]. It should be noted that widely different values have been reported for the rate of deactivation near 0 mV, approximately ranging from 10–50 s^−1^ [39, 69–71], which translates into a time constant of deactivation of 20–100 ms. Thus, our value of 50 ms is in line with these experimental data but somewhat uncertain.

## 3. State Diagrams of the *I*_*f*_ Channel

**Figure 2:**
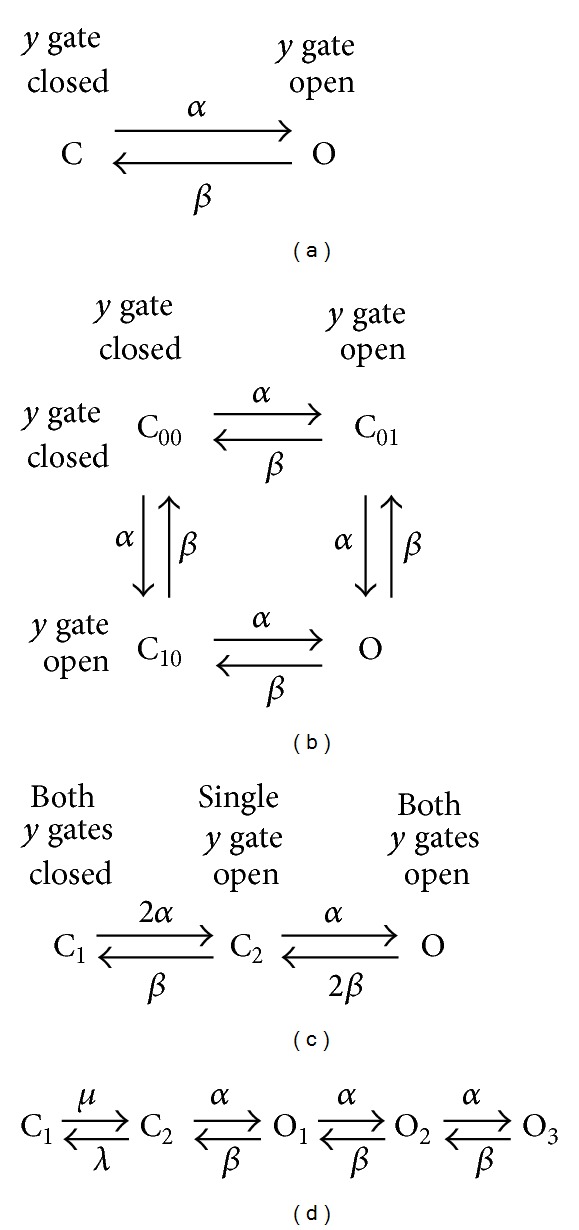
State diagrams of the *I*_*f*_ channel used in mathematical models of the hyperpolarization-activated current in rabbit sinoatrial node pacemaker cells. (a) Two-state model with a first-order Hodgkin and Huxley type *y* gate. The channel flips between its open state O and closed state C at rates *α* and *β*. (b) Hodgkin and Huxley type model with two identical *y* gates. (c) Three-state equivalent of the state diagram of (b). (d) Five-state model, with two closed states (C_1_ and C_2_) and three open states (O_1_, O_2_, and O_3_), as used in the mathematical model of a rabbit sinoatrial node pacemaker cell by Sarai et al. [78].

Several kinetic schemes have been proposed to describe the behavior of the *I*_*f*_ channel, varying from a simple two-state scheme as diagrammed in Figure 2(a) to a complex scheme with as many as five open and three closed configurations [72]. In mathematical models of *I*_*f*_ in rabbit SA nodal pacemaker cells—either as a model of *I*_*f*_ per se or as part of a model of the pacemaker activity of SA nodal myocytes—two-, three-, and five-state kinetic schemes have been used in relation to *I*_*f*_.

### 3.1. Two-State Model

In the two-state model (Figure 2(a)), the *I*_*f*_ channel flips between its open (conducting) state O and its closed (nonconducting) state C, controlled by a Hodgkin and Huxley [73] type *y* gate with voltage-dependent rate constants *α* and *β*. Accordingly, *I*_*f*_ is given by

(3) $$\begin{array}{c} I_{f}=y\times g_{f}\times \left(V_{m}-E_{f}\right), \end{array}$$

in which *g*_*f*_ is the fully activated *I*_*f*_ conductance, *E*_*f*_ is the  *I*_*f*_  reversal potential, and the gating variable *y*, with 0 ≤ *y* ≤ 1, obeys the first-order differential equation:

(4) $$\begin{array}{c} \frac{dy}{dt}=\alpha \times \left(1-y\right)-\beta \times y \end{array}$$

or, equivalently,

(5) $$\begin{array}{c} \frac{dy}{dt}=\frac{\left(y_{\infty }-y\right)}{\tau }, \end{array}$$

with

(6) $$\begin{array}{c} y_{\infty }=\frac{\alpha }{\left(\alpha +\beta \right)},\\ \tau =\frac{1}{\left(\alpha +\beta \right)}. \end{array}$$

In case of a voltage clamp step at time zero, the analytical solution to (4) becomes

(7) $$\begin{array}{c} y\left(t\right)=y_{\infty }-\left(y_{\infty }-y_{0}\right)\times \exp\left(-\frac{t}{\tau }\right). \end{array}$$

The gating variable *y* thus attains a new steady-state value *y*_*∞*_ in a monoexponential fashion with time constant *τ*.

The advantage of the two-state model is that it allows a direct translation of experimental data on *I*_*f*_, which are commonly acquired under voltage clamp conditions and presented in terms of a Boltzmann equation like that of Figure 1(c) and time constants of (de)activation, into a mathematical description through

(8) $$\begin{array}{c} \alpha =\frac{y_{\infty }}{\tau }, \end{array}$$

(9) $$\begin{array}{c} \beta =\frac{\left(1-y_{\infty }\right)}{\tau }. \end{array}$$

The two-state model has been used by DiFrancesco and Noble [70], Dokos et al. [74], and Zhang et al. [75]. 

### 3.2. Three-State Model

Experimentally, an initial delay or sigmoidal onset may be observed in both activation and deactivation of *I*_*f*_ under voltage clamp conditions. Following the approach by Hodgkin and Huxley [73] to describe the sigmoid course of activation of the potassium current in their nerve fibers, van Ginneken and Giles [39] introduced a second *y* gate in their mathematical description of *I*_*f*_ to account for the observed delay “semiquantitatively.” Accordingly, *I*_*f*_ is now given by

(10) $$\begin{array}{c} I_{f}=y^{2}\times g_{f}\times \left(V_{m}-E_{f}\right). \end{array}$$

The experimentally determined Boltzmann curve then corresponds with *y*_*∞*_^2^ rather than *y*_*∞*_, while determination of the rate constants *α* and *β* requires detailed analysis of the voltage clamp traces, as carried out by van Ginneken and Giles [39]. Unfortunately, they erroneously doubled their experimentally observed  *I*_*f*_  decay rate to obtain the decay rate of *y*, whereas they should have halved it, as set out in detail by Dokos et al. [74].

With two independent gates, there are four different states of the channel (Figure 2(b)). However, because the two gates are kinetically identical, the four-state scheme of Figure 2(b) can be simplified to the three-state scheme of Figure 2(c), in which the essentially identical states C_01_ and C_10_ of Figure 2(b) have been combined into a single state C_2_. The three-state model of Figure 2(c), with two independent, kinetically identical *y* gates, has been employed by Demir et al. [76], Kurata et al. [77], Maltsev and Lakatta [66], and Severi et al. [67].

Unlike the two-state model of Figure 2(a), the three-state model accounts for the sigmoidal onset of *I*_*f*_ activation that may be observed in voltage clamp traces. It should, however, be realized that this delay in activation shows a remarkable variability and may even be absent [38, 39]. The three-state model accounts for the initial delay in *I*_*f*_ activation, but not for any delay in deactivation, but such delay in deactivation is not observed in case of moderate and short hyperpolarizations as take place during normal SA nodal pacemaker activity [39].

### 3.3. Five-State Model

In many mathematical models of *I*_*f*_ in rabbit SA nodal pacemaker cells, a two- or three-state kinetic scheme is used [39, 66, 67, 70, 74–77]. In the mathematical model of an SA nodal pacemaker cell by Sarai et al. [78], however, a five-state scheme is used with two closed states (C_1_ and C_2_) and three open states (O_1_, O_2_, and O_3_), as diagrammed in Figure 2(d). This five-state scheme was introduced by Maruoka et al. [71] to describe *I*_*f*_ under voltage clamp conditions and included in the “Kyoto model,” which provides a common set of equations for ventricular myocytes and SA nodal pacemaker cells, by Matsuoka et al. [79] to account for the “sigmoidal onset of activation on hyperpolarization, and delayed removal of activation on depolarization beyond the reversal potential”.

## 4. Mathematical Models of *I*_*f*_

**Figure 3:**
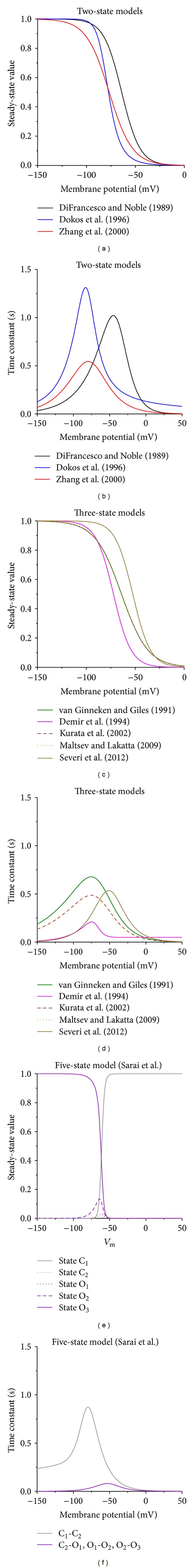
Kinetics of the *I*_*f*_ channel in mathematical models of the hyperpolarization-activated current in rabbit sinoatrial node pacemaker cells. ((a), (b)) Steady-state value (a) and time constant (b) of the gating variable *y* in the single-gate models by DiFrancesco and Noble [70], Dokos et al. [74], and Zhang et al. [75], as indicated. ((c), (d)) Steady-state value (c) and time constant (d) of the gating variable *y* in the two-gate models by van Ginneken and Giles [39], Demir et al. [76], Kurata et al. [77], Maltsev and Lakatta [66], and Severi et al. [67], as indicated. ((e), (f)) Steady-state values for each of the five states (e) and time constants for each of the four transitions (f) in the five-state model by Sarai et al. [78].

**Figure 4:**
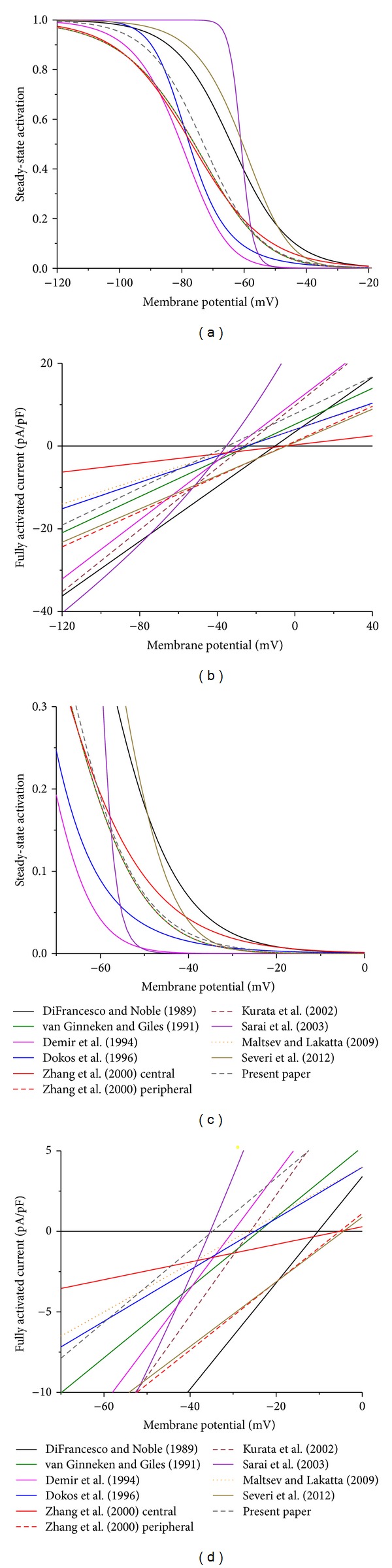
Steady-state *I*_*f*_ activation and fully activated current in 11 mathematical models of the hyperpolarization-activated current in rabbit sinoatrial node pacemaker cells. (a) Steady-state *I*_*f*_ activation. (b) Fully activated current. (c) Steady-state  *I*_*f*_ activation on expanded activation and membrane potential scales. (d) Fully activated current on expanded current and membrane potential scales.

Apart from differences in the kinetic schemes, the aforementioned models of *I*_*f*_ in rabbit SA nodal pacemaker cells differ in the associated rate constants as well as the conductance and reversal potential of *I*_*f*_, which together determine the course of *I*_*f*_ during an action potential, that is, under current clamp conditions. In this section, we present and discuss, in chronological order, the various models of *I*_*f*_ that we mentioned in the previous sections. Figures 3 and 4 and Table 1 summarize the main characteristics of the various models. At this point, the reader may want to consult Figures 3 and 4 and Table 1 and then move on to Section 5.

In several cases, the fully activated conductance and/or reversal potential of *I*_*f*_ were not explicitly stated in the model description and had to be determined from the relative sodium and potassium conductance of *I*_*f*_, in combination with the intracellular and extracellular sodium and potassium concentrations of the model cell through

(11) $$\begin{array}{c} g_{f}\times \left(V_{m}-E_{f}\right)=g_{f,\;\text{K}}\times \left(V_{m}-E_{\text{K}}\right)+g_{f,\;\text{Na}}\times \left(V_{m}-E_{\text{Na}}\right), \end{array}$$

where *g*_*f*, K_ and *g*_*f*, Na_ are the sodium and potassium conductance of *I*_*f*_, respectively, and *E*_K_ and *E*_Na_ are the Nernst potentials for sodium and potassium, respectively. Conversely, (11) can be used to estimate the *g*_*f*, Na_ : *g*_*f*, K_ ratio if *E*_*f*_ and the ion concentrations are known.

### 4.1. Model of DiFrancesco and Noble

**Figure 5:**
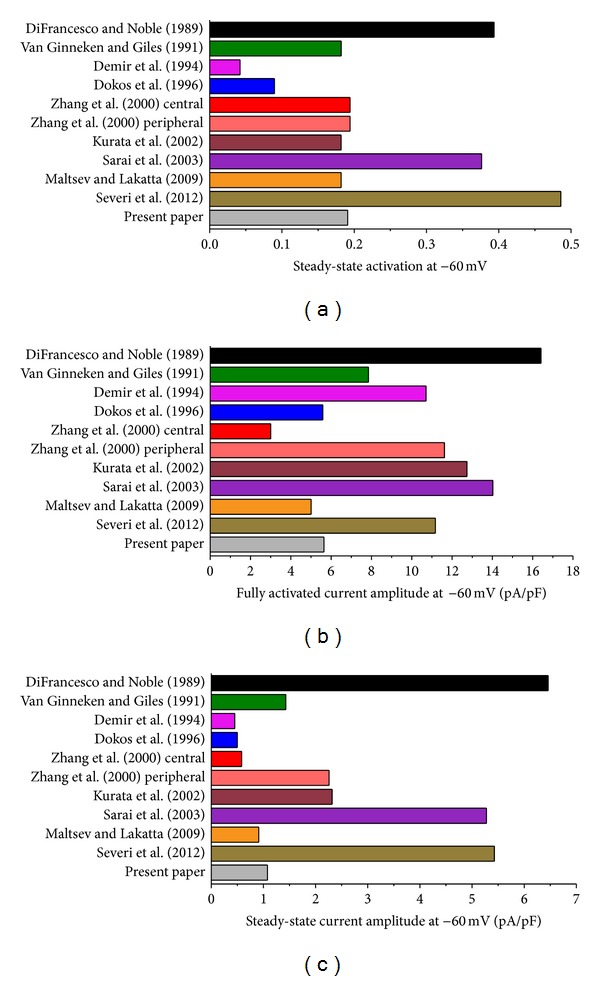
Steady-state *I*_*f*_ activation, fully activated current amplitude and steady-state current amplitude at −60 mV in 11 mathematical models of the hyperpolarization-activated current in rabbit sinoatrial node pacemaker cells. (a) Steady-state *I*_*f*_ activation. (b) Fully activated current amplitude. (c) Steady-state current amplitude.

**Figure 6:**
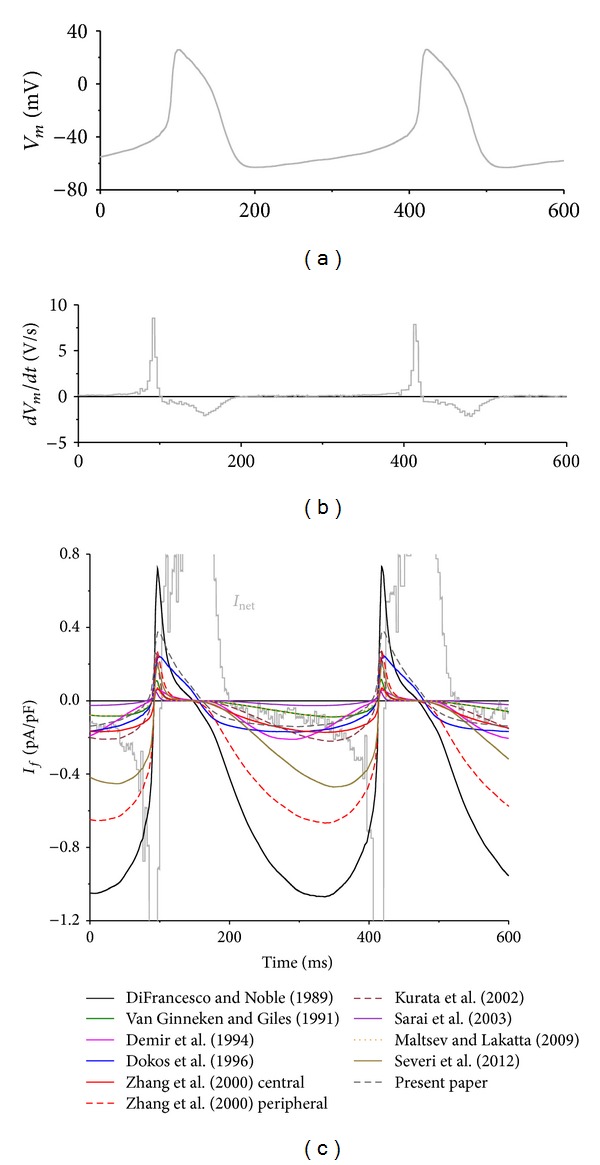
Numerical reconstruction of the time course of *I*_*f*_ during pacemaker activity of rabbit sinoatrial node cells. (a) Experimentally recorded action potentials of a single rabbit SA nodal pacemaker cell. (b) Associated time derivative (*dV*_*m*_/*dt*). (c) Associated time course of *I*_*f*_ as reconstructed using the membrane potential values of the recorded action potentials and mathematical models of this current in rabbit sinoatrial node pacemaker cells. Also shown is the net membrane current (*I*_net_), as derived from *I*_net_ = −*C*_*m*_ × *dV*_*m*_/*dt*, where *C*_*m*_ and *V*_*m*_ denote membrane capacitance and membrane potential, respectively.

The aim of DiFrancesco and Noble [70] was to provide a simple description of *I*_*f*_ “relevant to the reconstruction of the diastolic phase of the spontaneous action potential.” They noted that, for reconstruction purposes, it would be sufficient to describe the kinetics of *I*_*f*_ with a simple first-order Hodgkin and Huxley [73] type model and that, in this context, a more complex model was not justified. Thus, they adopted the two-state model of Figure 2(a) and (3)–(6) to describe  *I*_*f*_. From their experimental data on *I*_*f*_ in SA nodal pacemaker cells they derived

(12) $$\begin{array}{c} y_{\infty }=\frac{1}{\left\{1+\exp\left[0.10811\times \left(V_{m}+64\right)\right]\right\}}, \end{array}$$

(13) τ=1[exp⁡(−2.00084−0.03584×Vm)+exp⁡(2.4+0.08×Vm)]

as equations for *y*_*∞*_ and *τ* (in s), respectively, from which *α* and *β* can be derived through (8) and (9). The black curves in Figures 3(a) and 3(b), are constructed from (12) and (13), respectively.  Figure 3(a) shows the steady-state value of *y*, that is, *y*_*∞*_, for each of the two-state models discussed in Section 3, whereas Figure 3(b) shows the associated time constant for each of the models.

Figure 4(a) and, on expanded current and membrane potential scales, Figure 4(c) show the steady-state activation curve of *I*_*f*_ for each of the models discussed in Section 3. In addition, the curve that we determined experimentally (Figure 1(c)) is shown as a dark gray dashed curve. In case of the simple two-state model of DiFrancesco and Noble [70], the black curve of Figure 4(a) is identical to that of Figure 3(a). Figure 4(a) illustrates that the steady-state activation curve of DiFrancesco and Noble [70] is similar in shape to the curve that we presented in Section 2—Boltzmann curves with slope factors of 9.2 and 9.0, respectively—but that the half-activation voltage is more positive (−64 versus −73 mV, as listed in Table 1).

In their description of  *I*_*f*_, DiFrancesco and Noble [70] used a reversal potential of −10.3 mV and a fully activated conductance of 9909 pS, that is, 0.3303 nS/pF when normalized to their membrane capacitance of 30 pF. Thus, (3) reads

(14) $$\begin{array}{c} I_{f}=y\times 0.3303\times \left(V_{m}+10.3\right), \end{array}$$

with *V*_*m*_ expressed in mV and *I*_*f*_ in pA/pF. This yields the fully activated current that appears as a black line in Figure 4(b) and, on expanded current and membrane potential scales, in Figure 4(d). As can be appreciated from Figures 4(b) and 4(d), the fully activated conductance of 0.3303 nS/pF is *≈*50% larger and the reversal potential of −10.3 mV is *≈*25 mV more positive than in our experimental data.

### 4.2. Model of van Ginneken and Giles

van Ginneken and Giles [39] carried out a comprehensive study on  *I*_*f*_  in isolated rabbit SA nodal pacemaker cells. They analyzed their experimental data in terms of the three-state kinetic scheme of Figure 2(c) and arrived at

(15) $$\begin{array}{c} y_{\infty }=\frac{1}{\left\{1+\exp\left[\left(V_{m}+64\right)/13.5\right]\right\}} \end{array}$$

for the steady-state value of* y*, with *V*_*m*_ expressed in mV, and at

(16) $$\begin{array}{c} \alpha =\exp\left[-0.0220741\times \left(V_{m}+386.9\right)\right],\\ \beta =\exp\left[0.052\times \left(V_{m}-73.08\right)\right] \end{array}$$

for the associated rate constants, both expressed in ms^−1^, which can be turned into *y*_*∞*_ and *τ* for display in Figures 3(c) and 3(d) (green curves), through (6). The steady-state activation curve of  *I*_*f*_  can be obtained by squaring *y*_*∞*_ and is shown in Figures 4(a) and 4(c). In the physiological membrane potential range, it is highly similar to our Boltzmann fit of Figure 1(c), which appears in Figures 4(a) and 4(c) as a dark gray dashed curve.

In their experiments, van Ginneken and Giles [39] observed mean values of −24 mV and 12.0 nS for the reversal potential and fully activated conductance of *I*_*f*_, respectively. When normalized to their mean membrane capacitance of 55 pF, the fully activated conductance becomes 0.2182 nS/pF, which is remarkably similar to the value of 0.224 nS/pF that we determined from our experiments (Figure 1). With these values, (10) reads

(17) $$\begin{array}{c} I_{f}=y^{2}\times 0.2182\times \left(V_{m}+24\right), \end{array}$$

with *V*_*m*_ expressed in mV and *I*_*f*_ in pA/pF. This yields the fully activated current that appears as a green line in Figures 4(b) and 4(d). The reversal potential of −24 mV is more positive than we observed, but this may reflect differences in the bath and pipette solutions and the extent to which the data were corrected for the liquid junction potential.

Unfortunately, as already mentioned in Section 3.2, van Ginneken and Giles [39] erroneously doubled their experimentally observed decay rate in their analysis instead of halving it. This affects *β* and *τ*, but not *y*_*∞*_. Furthermore, it should be noted that all experiments were carried out at a fixed temperature between 30 and 33°C (±1°C), which may have led to an underestimation of the rates of activation and deactivation.

### 4.3. Model of Demir et al

In their mathematical model of an SA nodal pacemaker cell, Demir et al. [76] based their equations for *I*_*f*_ on the data by van Ginneken and Giles [39]. They also used the three-state model of Figure 2(c), but reanalyzed the raw data of van Ginneken and Giles [39], validating their equations in a comparison of model-generated voltage clamp traces with those reported by van Ginneken and Giles [39]. Furthermore, they converted the thus obtained time constant values to a temperature of 37°C, assuming a Q_10_ of 2.3. This reanalysis led to

(18) $$\begin{array}{c} y_{\infty }=\frac{1}{\left\{1+\exp\left[\left(V_{m}+72.2\right)/9\right]\right\}}, \end{array}$$

(19) τ={1.6483×exp⁡[−(Vm+54.06)24.33]    +14.01055{0.7+exp⁡[−(Vm+60)/5.5]}}−1

as equations for *y*_*∞*_ and *τ* (in s), respectively. The thus defined curves are shown in Figures 3(c) and 3(d) and are remarkably different from those based on the equations by van Ginneken and Giles [39]. This also holds for the steady-state activation curve of *I*_*f*_ obtained by squaring *y*_*∞*_, which is shown in Figures 4(a) and 4(c). 

In their model, Demir et al. [76] selected an *I*_*f*_ reversal potential of −30 mV. For the fully activated conductance, they chose of value of 19.63 nS, which turns into a value of 0.3569 nS/pF when normalized to the model's membrane capacitance of 55 pF, which Demir et al. [76] based on the mean value reported by van Ginneken and Giles [39]. With these values, (10) reads

(20) $$\begin{array}{c} I_{f}=y^{2}\times 0.3569\times \left(V_{m}+30\right), \end{array}$$

with *V*_*m*_ expressed in mV and *I*_*f*_ in pA/pF. The associated fully activated current is shown in Figures 4(b) and 4(d).

### 4.4. Model of Dokos et al

In 1996, two years after Demir et al. [76], Dokos et al. [74] also published a mathematical model of an SA nodal pacemaker cell. However, they used the simpler first-order Hodgkin and Huxley type model of Figure 2(a). As Demir et al. [76], Dokos et al. [74] also based their equations for the kinetics of *I*_*f*_ on the raw data by van Ginneken and Giles [39], arriving at

(21) $$\begin{array}{c} \alpha =\frac{0.36\times \left(V_{m}+137.8\right)}{\left\{\exp\left[0.066\times \left(V_{m}+137.8\right)\right]-1\right\}},\\ \beta =\frac{0.1\times \left(V_{m}+76.3\right)}{\left\{1-\exp\left[-0.21\times \left(V_{m}+76.3\right)\right]\right\}} \end{array}$$

for the rate constants *α* and *β*, both expressed in s^−1^, which have been turned into *y*_*∞*_ and *τ* for display in Figures 3(a) and 3(b) (blue curves), using (6). The steady-state activation curve is shown in Figures 4(a) and 4(c) and differs not only from that of van Ginneken and Giles [39] but also from that of Demir et al. [76].

Dokos et al. [74] selected values of the sodium and potassium conductance of *I*_*f*_ “to produce a reversal potential of −25 mV and a cycle length prolongation of *≈*27% in the free-running model when *I*_*f*_ was abolished.” The resulting reversal potential is −24.97 mV, and the resulting fully activated conductance is 5.102 nS, which turns into a value of 0.1595 nS/pF when normalized to the model's membrane capacitance of 32 pF, which Dokos et al. [74] adopted from the early model by Wilders et al. [80]. With these values, (3) reads

(22) $$\begin{array}{c} I_{f}=y\times 0.1595\times \left(V_{m}+24.97\right), \end{array}$$

with *V*_*m*_ expressed in mV and *I*_*f*_ in pA/pF. The associated fully activated current is shown in Figures 4(b) and 4(d).

### 4.5. Model of Zhang et al

Zhang et al. [75] also used a single-gate description of *I*_*f*_, as in Figure 2(a), in the mathematical model of an SA nodal pacemaker cell that they published in 2000. They created two versions, one for a small cell, with a membrane capacitance of 20 pF, presumably originating from the center of the SA node, and one for a large cell, with a membrane capacitance of 65 pF, presumably originating from the periphery of the SA node. They introduced rate constants *α* and *β*, both in s^−1^, given by

(23) $$\begin{array}{c} \alpha =\exp\left[-\frac{V_{m}+78.91}{26.62}\right],\\ \beta =\exp\left[\frac{V_{m}+75.13}{21.25}\right], \end{array}$$

which they validated in a comparison with the experimental data for *y*_*∞*_ and *τ* of van Ginneken and Giles [39] and Liu et al. [81]. The associated *y*_*∞*_ and *τ* are shown in Figures 3(a) and 3(b) (red curves) and the steady-state activation curve in Figures 4(a) and 4(c). These hold for both versions of the model.

Zhang et al. [75] assumed identical sodium and potassium conductance values for *I*_*f*_. This results in a reversal potential of −5.25 mV, which differs from our experimentally observed value (Figure 1(b)) by 30 mV. The fully activated conductance of *I*_*f*_ was validated against the current-voltage relationships reported by Honjo et al. [82], who found that the fully activated current density of *I*_*f*_ (in pA/pF) of SA nodal cells increases with the membrane capacitance of the cells. Zhang et al. [75] selected a fully activated *I*_*f*_ conductance of 1.096 nS for their central cell model and 13.8 nS for their peripheral cell model. Normalized to the membrane capacitance of 20 and 65 pF, these values are 0.0548 and 0.2123 nS/pF, respectively. With these values, (3) reads

(24) $$\begin{array}{c} I_{f}=y\times 0.0548\times \left(V_{m}+5.25\right), \end{array}$$

for the central cell model and

(25) $$\begin{array}{c} I_{f}=y\times 0.2123\times \left(V_{m}+5.25\right), \end{array}$$

for the peripheral cell model, both with *V*_*m*_ expressed in mV and *I*_*f*_ in pA/pF. The associated fully activated current is shown as a red solid line for the central cell model and a dashed line for the peripheral cell model in Figures 4(b) and 4(d).

### 4.6. Model of Kurata et al

In 2002, Kurata et al. [77] published “an improved mathematical model for a single pacemaker cell of the rabbit SA node.” This primary cell model has a membrane capacitance of 32 pF, in line with the earlier models by Wilders et al. [80] and Dokos et al. [74]. The formulation for *I*_*f*_ was adopted from the model of Wilders et al. [80], who had in turn used the equations and parameters of van Ginneken and Giles [39] and arrived on potassium and sodium conductances of 7.4 and 4.6 nS, respectively, for *I*_*f*_, based on the observed reversal potential of −24 mV. Thus, *y*_*∞*_ is given by (15), which explains that the *y*_*∞*_ curve in Figure 3(c) and the steady-state activation curve in Figures 4(a) and 4(c), of Kurata et al. [77] and van Ginneken and Giles [39], coincide.

The equation for the time constant *τ* was derived from the rate constants of (16) but corrected for 37°C by the use of a Q_10_ factor of 2.3 following Demir et al. [76], thus arriving at

(26) τ=0.71665{exp⁡[−(Vm+386.9)/45.3]+exp⁡[(Vm−73.08)/19.23]},

in which *τ* is in ms and 0.71665 is the correction factor for the temperature of 30–33°C in the experiments of van Ginneken and Giles [39]. Thus, the Kurata et al. [77] curve in Figure 3(d) is similar in shape but smaller in magnitude than the van Ginneken and Giles [39] curve.

For the fully activated conductance of  *I*_*f*_, Kurata et al. [77] used the value of 12 nS of van Ginneken and Giles [39], which, with their membrane capacitance of 32 pF, results in a normalized value of 0.375 nS/pF in the equation for *I*_*f*_:

(27) $$\begin{array}{c} I_{f}=y^{2}\times 0.375\times \left(V_{m}+26.02\right), \end{array}$$

in which *I*_*f*_ is again expressed in pA/pF and *V*_*m*_ in mV. The reversal potential of −26.02 mV deviates from the value of −24 mV of the Wilders et al. [80] model, because there are some minor differences in sodium and potassium concentrations between the models. The fully activated current is shown in Figures 4(b) and 4(d).

### 4.7. Model of Sarai et al

The SA nodal cell model of Sarai et al. [78] is part of the “Kyoto model” that was introduced by Matsuoka et al. [79] in 2003. As detailed in Section 3.3 and diagrammed in Figure 2(d), the *I*_*f*_ channel has two closed states (C_1_ and C_2_) and three open states (O_1_, O_2_, and O_3_). The rate constants *μ* and *λ* that determine the rate of transitions between the two closed states C_1_ and C_2_ are given by

(28) $$\begin{array}{c} \mu =\frac{1}{\left[4.5\times 10^{7}\times \exp\left(V_{m}/8\right)+500\times \exp\left(V_{m}/200\right)\right]},\\ \lambda =\frac{1}{\left[10.5\times \exp\left(-V_{m}/16.4\right)+0.4\times \exp\left(-V_{m}/400\right)\right]}, \end{array}$$

where *μ* and *λ* are both in ms^−1^ and *V*_*m*_ in mV. The remaining three transitions (see Figure 2(d)) are all controlled by rate constants *α* and *β* given by

(29) $$\begin{array}{c} \alpha =\frac{1}{\left[3500\times \exp\left(V_{m}/16.8\right)+0.3\times \exp\left(V_{m}/400\right)\right]},\\ \beta =\exp\left[\frac{V_{m}+75.13}{21.25}\right], \end{array}$$

where *α* and *β* are both in ms^−1^ and *V*_*m*_ in mV. Under steady-state conditions, most of the *I*_*f*_ channels are either in the closed state C_1_ or in the open state O_3_, as illustrated in Figure 3(e). The time constants associated with *μ* and *λ* and with *α* and *β* are both shown in Figure 3(f), which demonstrates that the transition between C_1_ and C_2_ is relatively slow and that the other transitions are relatively fast. The steady-state activation of *I*_*f*_ can be computed from the fraction of *I*_*f*_ channels in each of the open states O_1_, O_2_, and O_3_ as shown in Figure 3(e). This yields the curve shown in Figures 4(a) and 4(c), which is strikingly steep when compared to each of the other curves. 

Unlike the other models, the fully activated current *I*_*f*,full_ is not ohmic and thus linearly dependent on the membrane potential but determined by

(30) $$\begin{array}{c} I_{f,\text{full}}=1.821\times {\text{CF}}_{\text{Na}}+7.7286\times {\text{CF}}_{\text{K}}, \end{array}$$

in which 1.821 and 7.7286 are the permeabilities for Na^+^ and K^+^ in pA/mM, and CF_Na_ and CF_K_ are given by the “constant-field equations”

(31) CFNa={Vm/(RT/F)}×{[Na+]i−[Na+]e×exp⁡(−Vm/(RT/F))}{1−exp⁡⁡(−Vm/(RT/F))},CFK={Vm/(RT/F)}×{[K+]i−[K+]e×exp⁡(−Vm/(RT/F))}{1−exp⁡(−Vm/(RT/F))},

in which [Na^+^]_i_, [Na^+^]_e_, [K^+^]_i_, and [K^+^]_e_ are the intracellular and extracellular concentrations of Na^+^ and K^+^ in mM, and the fraction *RT*/*F* is determined by the universal gas constant *R*, the absolute temperature *T*, and the Faraday constant *F* and amounts to 26.7 mV. Using (30)-(31) and the model concentrations of 140 mM for [Na^+^]_e_, 5.4 mM for [K^+^]_e_, *≈*4.6 mM for [Na^+^]_i_, and *≈*143 mM for [K^+^]_i_, one obtains the fully activated current as shown in Figures 4(b) and 4(d), with a reversal potential of −35.3 mV. In the membrane potential range from −65 to −40 mV, the curve is almost linear with a conductance of 0.5–0.6 nS/pF.

### 4.8. Model of Maltsev and Lakatta

In 2009, Maltsev and Lakatta [66] published a mathematical model of an SA nodal pacemaker cell that is based on the model by Kurata et al. [77] but incorporates a submembrane “calcium clock” that interacts with the “membrane clock” in producing the pacemaker activity of the model cell [6, 9]. Maltsev and Lakatta [66] adopted the *I*_*f*_ kinetics of Kurata et al. [77]. This explains why the Maltsev and Lakatta [66] and Kurata et al. [77] curves in Figures 3(c) and 3(d) and Figures 4(a) and 4(c) coincide.

Maltsev and Lakatta [66] reduced the fully activated *I*_*f*_ conductance of Kurata et al. [77] by 60%, from 0.375 to 0.15 nS/pF. Furthermore, their (fixed) ion concentrations of 10, 140, 140, and 5.4 mM for [Na^+^]_i_, [Na^+^]_e_, [K^+^]_i_, and [K^+^]_e_, respectively, are slightly different from the concentrations in the model by Kurata et al. [77], resulting in an *I*_*f*_ reversal potential of −26.62 mV. Thus, the line representing the fully activated current of Maltsev and Lakatta [66] in Figures 4(b) and 4(d) is less steep and shifted by 0.6 mV compared to the line obtained from the Kurata et al. [77] model.

### 4.9. Model of Severi et al

The most recent mathematical model of a rabbit SA nodal pacemaker cell is that of Severi et al. [67], which they presented as “an updated computational model of rabbit sinoatrial action potential to investigate the mechanisms of heart rate modulation.” In this model, the kinetic and conductive properties of *I*_*f*_ are largely based on the work of DiFrancesco and Noble from the 1980s. The kinetics are adopted from the early SA nodal cell model by Noble et al. [83], who used a Hodgkin and Huxley type model with two identical *y* gates, as in Figures 2(b) and 2(c). However, Severi et al. [67] shifted the associated *y*_*∞*_ and *τ* curves to more depolarized potentials by *≈*11 mV, based on experimental data from Altomare et al. [84] and Barbuti et al. [85], producing

(32) y∞=1{1+exp⁡[(Vm+52.5)/9]},τ=0.7{0.0708×exp⁡[−(Vm+5.0)/20.28]+10.6×exp⁡[Vm/18]},

where *V*_*m*_ is in mV and *τ* in s. The *y*_*∞*_ and *τ* curves are shown in Figures 3(c) and 3(d) and the steady-state activation curve in Figures 4(a) and 4(c).

According to the original description by DiFrancesco and Noble [86] and in line with Noble et al. [83], Severi et al. [67] assumed identical conductance values for the sodium and potassium components of *I*_*f*_, with a total conductance of 6.429 nS. Because, as in the models by Wilders et al. [80], Dokos et al. [74], and Maltsev and Lakatta [66], the model cell has a membrane capacitance of 32 pF, the normalized *I*_*f*_ conductance amounts to 0.2009 nS/pF (Table 1). With the model concentrations for sodium and potassium ions, the *I*_*f*_ reversal potential amounts to −4.39 mV, which is 7.9 mV more positive than in the model by Noble et al. [83], due to differences in ion concentrations between the two models. Thus, *I*_*f*_ is given by

(33) $$\begin{array}{c} I_{f}=y^{2}\times 0.2009\times \left(V_{m}+4.39\right), \end{array}$$

in which *I*_*f*_ is expressed in pA/pF and *V*_*m*_ in mV and which yields the fully activated current shown in Figures 4(b) and 4(d).

### 4.10. Novel Model

Apart from the ten *I*_*f*_ models detailed in Sections 4.1–4.9, Figures 3 and 4 show curves labeled “present paper.” These curves represent a novel model for *I*_*f*_ based on the experimental data that we presented in Section 2. We use the kinetic scheme of Figure 2(a) and describe *I*_*f*_ by

(34) $$\begin{array}{c} I_{f}=y\times 0.224\times \left(V_{m}+34.8\right), \end{array}$$

with *y*_*∞*_ and *τ* given by

(35) y∞=1{1+exp⁡[(Vm+73)/9]},τ=0.05+[75.8×exp⁡(0.083×Vm)      +0.0233×exp⁡(−0.043×Vm)]−1,

where  *I*_*f*_ is in pA/pF, *V*_*m*_ is in mV, and *τ* in s.

As can be appreciated from Figure 4(c), the steady-state activation curve of our model closely matches with that of van Ginneken and Giles [39], Kurata et al. [77], and Maltsev and Lakatta [66] in the physiological membrane potential range, whereas there are significant discrepancies with those of other models, in particular the models by DiFrancesco and Noble [70], Sarai et al. [78], and Severi et al. [67]. The *I*_*f*_ conductance of 0.224 nS/pF, on the other hand, closely matches the values of 0.2182, 0.2123, and 0.2009 nS/pF of the models by van Ginneken and Giles [39], Zhang et al. [75] (peripheral cell), and Severi et al. [67], respectively, as can be appreciated from the slope of the lines in Figure 4(d). However, Figure 4(d) also illustrates that the *I*_*f*_ reversal potential of the latter two models differs from that of our model by as much as 30 mV, which creates an almost twofold difference in *I*_*f*_ driving force near the maximum diastolic potential of an SA nodal action potential.

Our model does not have an explicit cAMP dependence. However, autonomic modulation of *I*_*f*_ can be incorporated through a shift of the steady-state activation curve along the voltage axis, by up to *≈* 10 mV in the positive direction for adrenergic modulation and up to *≈*10 mV in the negative direction for cholinergic modulation. Such shift has been observed experimentally [11, 87] and has been incorporated in several models, for example, [88–92], to reflect the autonomic modulation of *I*_*f*_ through acetylcholine and (nor) adrenalin. In addition, a similar voltage shift should be applied to the time constant curve to account for the experimentally observed cAMP dependence of this curve [93]. The latter shift has been ignored in most models, but not in the recent model of Severi et al. [67].

## 5. Reconstruction of *I*_*f*_

In the previous section, we have identified models of *I*_*f*_ in terms of characteristics derived from and related to voltage clamp experiments, including rate constants, steady-state activation, fully activated conductance, and reversal potential. In the present section, we show how these characteristics determine the course of *I*_*f*_ during an action potential, that is, under current clamp conditions.

### 5.1. Steady-State Current

Before reconstructing *I*_*f*_ during an action potential in Section 5.2, we use the data of Figure 4 to compute the steady-state current at −60 mV to get an impression of the amplitude of *I*_*f*_ that one might expect for each of the models.  Figure 5(a) shows the steady-state activation at −60 mV in each of the 11 models. This steady-state activation ranges from 0.042 in the Demir et al. [76] model to 0.486, that is, almost 50% activation of *I*_*f*_ at −60 mV, in the Severi et al. [67] model, which constitutes an almost 12-fold difference. Most models, including the one based on our experimental data, predict a value near 19% for the steady-state activation at −60 mV (Figure 5(a)).

As for the steady-state activation, there is a wide difference in the fully activated current amplitude at −60 mV, which is not only determined by the *I*_*f*_ conductance but also by the *I*_*f*_ reversal potential as a determinant of the driving force. With a value of 16.4 pA/pF, the model by DiFrancesco and Noble [70] has the largest amplitude, whereas the central cell model by Zhang et al. [75] shows the smallest amplitude with a value of 3.0 pA/pF, a 5.5-fold difference.

In combination, the steady-state activation of Figure 5(a) and the fully activated current amplitude of Figure 5(b) determine the amplitude of *I*_*f*_ that can be activated at −60 mV. We multiplied the fully activated current amplitude of Figure 5(b) by the steady-state activation of Figure 5(a) to arrive at the steady-state current amplitude of Figure 5(c). With values of 6.5, 5.3, and 5.4 pA/pF, respectively, the models by DiFrancesco and Noble [70], Sarai et al. [78], and Severi et al. [67] show a remarkably large amplitude. The smallest amplitude is obtained with the models of Demir et al. [76], Dokos et al. [74], and Zhang et al. [75] (central cell), with values of 0.45, 0.50, and 0.58 pA/pF, respectively. Overall, there is a >14-fold range in *I*_*f*_ that can be activated at −60 mV according to the models of Figure 5(c). With values of 0.91 and 1.08 pA/pF, respectively, the model by Maltsev and Lakatta [66] and the model based on our experimental data show similar values.

### 5.2. Dynamics of *I*_*f*_

**Figure 7:**
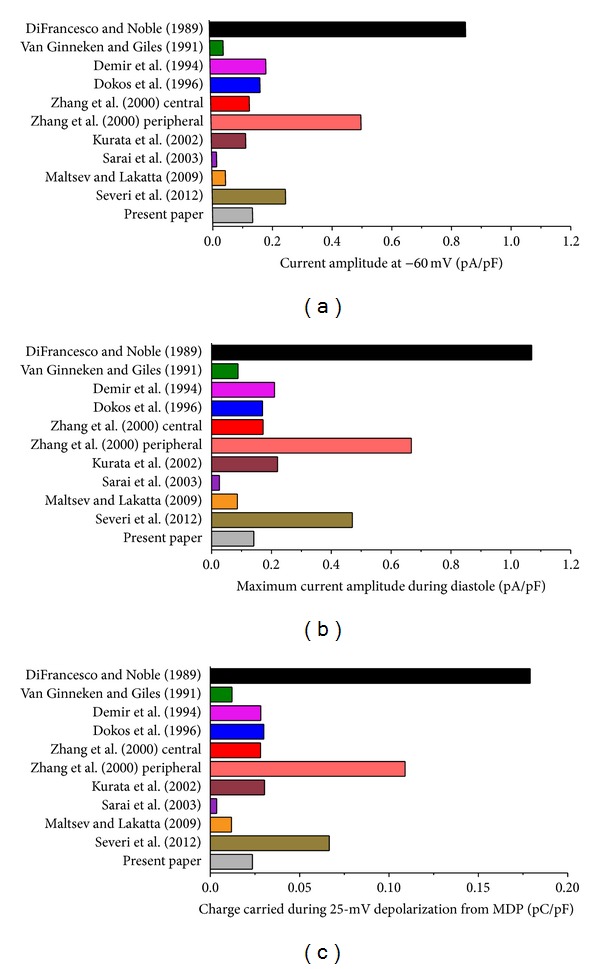
Diastolic *I*_*f*_ current amplitude at −60 mV, maximum *I*_*f*_ current amplitude during diastole, and charge carried by *I*_*f*_ during diastolic depolarization for each of the 11 reconstructed *I*_*f*_ current traces of Figure 6(c). (a) Diastolic *I*_*f*_ current amplitude at −60 mV. (b) Maximum *I*_*f*_ current amplitude during diastole. (c) Charge carried by *I*_*f*_ during the 25-mV, 200-ms diastolic depolarization from the maximum diastolic potential of −63 mV to −38 mV.

Figure 5 shows that the amount of *I*_*f*_ that can be activated at −60 mV varies widely between the models, but this does not imply that this is also the case during the course of action potential. In the latter case, the rate at which *I*_*f*_ activates and deactivates plays an important role. Therefore, we subjected each of the models to an “action potential clamp”: we reconstructed *I*_*f*_ during the experimentally recorded action potentials of Figure 6(a). These action potentials were applied as part of a sufficiently long train, and *I*_*f*_ was computed according to the equations listed in Section 4. The resulting *I*_*f*_ traces are shown in Figure 6(c), together with the net membrane current, *I*_net_, which was computed from the time derivative (*dV*_*m*_/*dt*) of the membrane potential trace of Figure 6(a), as shown in Figure 6(b). From the current traces shown in Figure 6(c), we computed the diastolic  *I*_*f*_ current amplitude at −60 mV (Figure 7(a)) as well as the maximum *I*_*f*_ current amplitude during diastole (Figure 7(b)). Also, we computed the charge carried by *I*_*f*_ during the 200 ms, 25 mV diastolic depolarization from the maximum diastolic potential of −63 mV to −38 mV (Figure 7(c)). Both Figure 6(c) and Figure 7(a) demonstrate that only a fraction of the steady-state current of Figure 5(c) is activated during an action potential. This fraction varies from 0.3% for the Sarai et al. model [78] to 42% for the Demir et al. model [76].

Figure 7(c) shows that, in the absence of other inward or outward membrane currents, the charge carried by  *I*_*f*_ during the 200 ms depolarization would be sufficient or almost sufficient to depolarize the membrane by the observed 25 mV. For example, our model based on the experimental data of Section 2 predicts a charge carried by  *I*_*f*_ of 0.024 pC/pF, which is equivalent to a depolarization of 24 mV. Notably, the models of Maltsev and Lakatta [66] and Severi et al. [67] predict depolarizations of 12 and 67 mV, respectively.

With a peak inward current of only 0.027 pA/pF (Figures 6(c) and 7(b)), the smallest *I*_*f*_ is obtained with the Sarai et al. model [78], although a relatively large *I*_*f*_ can be activated under steady-state conditions (Figure 5(c)). This emphasizes the important role of the rate at which *I*_*f*_ activates and deactivates, but in this particular case the exceptional steepness of the steady-activation curve (Figures 4(a) and 4(c)) also plays an important role. The importance of the rate of (de)activation is perhaps better illustrated with the Zhang et al. [75] peripheral and Kurata et al. [77] models that show a similar amplitude in Figure 5(c) but clearly different peak inward currents of 0.68 pA/pF for the Zhang et al. [75] peripheral cell model and threefold less, 0.23 pA/pF, for the Kurata et al. [77] model (Figures 6(c) and 7(b)). Of note, this cannot be explained by the use of a single-gate kinetic scheme by Zhang et al. [75] versus a double-gate scheme by Kurata et al. [77], as the effects of the selection of a single or double *y* gate on the reconstructed *I*_*f*_ are minimal [58, 83]. Rather, it may reflect the erroneously overestimated deactivation rate of  *I*_*f*_ (see Sections 3.2 and 4.2) in the Kurata et al. [77] model, which also affects the traces obtained with the related models by van Ginneken and Giles [39] and Maltsev and Lakatta [66]. The latter two traces almost coincide despite the smaller *I*_*f*_ conductance in the Maltsev and Lakatta [66] model. Here, the faster kinetics of the Maltsev and Lakatta [66]  *I*_*f*_ compensate for this smaller conductance.

In most models, *I*_*f*_ deactivates almost instantaneously at depolarized potentials, resulting in almost complete deactivation of *I*_*f*_ near the overshoot of the action potential and a slowly developing*I*_*f*_ during the subsequent diastolic phase. However, as set out in Section 2, *I*_*f*_ deactivation is fast but certainly not instantaneous at depolarized potentials. If this is taken into account, as in our novel model, *I*_*f*_ is available early in diastole and of relatively constant amplitude during diastolic depolarization. Interestingly, Zaza et al. [94] already noted that the presence of measurable inward 2 mM Cs^+^ sensitive current almost immediately after repolarization in their action potential clamp experiments on rabbit SA nodal cells is apparently at odds with the slow kinetics of *I*_*f*_ activation at diastolic potentials, that this suggests that *I*_*f*_ may not deactivate completely during repetitive activity, and that this would also increase the amount of *I*_*f*_ available during diastolic depolarization.

## 6. Conclusion

The various mathematical descriptions of  *I*_*f*_ that have been used in computer simulations show strikingly different characteristics when reconstructing the course of *I*_*f*_ during an action potential. This explains—at least to some extent—that some successfully use computer simulations to support their view that *I*_*f*_ plays a fundamental role in the generation of pacemaker activity and its rate control, while others provide computer simulation results in favor of their view that the role of *I*_*f*_ is limited to a modest contribution to rate control. We have identified some important caveats regarding the reconstruction of the course of *I*_*f*_ during an action potential through computer simulations. An obvious one is the use of appropriate activation kinetics and an appropriate value for the  *I*_*f*_ conductance. The half-activation voltage and fully activated conductance of most models, with values of −80 to −60 mV and *≈*0.2 nS/pF, respectively, are in line with the experimental data. However, the steep steady-state activation curve of the complex five-state model of Sarai et al. [78] is clearly at odds with the experimental data. A somewhat less obvious caveat is the selection of an *I*_*f*_ reversal potential that is in line with the experimental data. This reversal potential should be around −30 mV, but this is definitely not the case for the models of DiFrancesco and Noble [70], Zhang et al. [75], and Severi et al. [67]. The final and most important caveat to be taken into account is that *I*_*f*_ deactivation is not almost instantaneous at depolarized potentials. In most models, including the recent models by Maltsev and Lakatta [66] and Severi et al. [67], this deactivation is much faster than can be deduced from the scarce experimental data, which likely results in an underestimation of *I*_*f*_ during diastolic depolarization. Our novel model for the reconstruction of *I*_*f*_ in mathematical models of SA nodal pacemaker cells is simple and straightforward but takes care of all of these caveats.

## Figures and Tables

**Table 1:** Characteristics of the hyperpolarization-activated current (*I*_*f*_).

	*g*_*f*_ (nS/pF)	*E*_*f*_ (mV)	*g*_*f*,Na_ : *g*_*f*,K_ (ratio)	*V*_0.5_ (mV)	τ_−10_ (ms)	τ_+20_ (ms)
Experimental data						
DiFrancesco and Noble [70]	*≈*0.33	−10 to −20	—	*≈*−64	*≈*87	*≈*33
van Ginneken and Giles [39]	0.22 ± 0.02	−24 ± 2	—	*≈*−76	*≈*180	—
Present paper	0.22	−35	0.491	−73	71 ± 6	—
Mathematical models						
DiFrancesco and Noble [70]	0.3303	−10.3	—	−64	195	18
van Ginneken and Giles [39]	0.2182	−24	—	−76	38	8
Demir et al. [76]	0.3569	−30	0.524	−80	25	25
Dokos et al. [74]	0.1595	−25	0.600	−78	151	104
Zhang et al. [75] central	0.0548	−5	1.000	−77	47	11
Zhang et al. [75] peripheral	0.2123	−5	1.000	−77	47	11
Kurata et al. [77]	0.3750	−26	0.622	−76	27	6
Sarai et al. [78]	0.5–0.6	−35	0.65–0.91	−61	10	3
Maltsev and Lakatta [66]	0.1500	−27	0.622	−76	27	6
Severi et al. [67]	0.2009	−4	1.000	−60	57	11
Present paper	0.2240	−35	0.491	−73	80	53

*g*_*f*_: fully activated conductance; *E*_*f*_: reversal potential; *g*_*f*,Na_ : *g*_*f*,K_: ratio of sodium and potassium conductance; *V*_0.5_: half-activation voltage; *τ*_−10_ and *τ*_+20_: time constant of deactivation at −10 and +20 mV, respectively.

Experimental data are mean ± SEM. Data of DiFrancesco and Noble [70] and van Ginneken and Giles [39] are not corrected for liquid junction potential.
